# The Geometric Osteotomy: Joint Preservation in Juxta-Articular Surface Bone Neoplasms

**DOI:** 10.1080/13577149778263

**Published:** 1997-12

**Authors:** Eric L. Masterson, Riccardo Ferracini, Aileen M. Davis, Jay S. Wunder, Robert S. Bell

**Affiliations:** 1 University Musculoskeletal Oncology Unit Mount Sinai Hospital 600 University Avenue Suite 476 Toronto M5G 1X5 Canada; 2 University of Toronto Toronto Canada; 3 Princess Margaret Comprehensive Cancer Centre Toronto Canada

## Abstract

*Purpose*. To present the oncologic and functional results of a consecutive series of patients treated by geometric osteotomy and allograft reconstruction for juxta-articular surface bone neoplasms.

*Patients*. Twelve consecutive patients (mean age 28 years) underwent excision of benign aggressive or malignant juxta-articular surface bone neoplasms. In each case, only part of the circumference of the bone was excised, and the joint surface was preserved and kept in continuity with the remainder of the bone. The defects were filled with allograft or autograff, and internally fixed as appropriate.

*Methods*. Patients were eligible for the study if they had a histologically proven, primary tumour of bone adjacent to a joint such that the turnout could be completely excised with a partial cortical resection and preservation of the joint. The database at the University Musculoskeletal Oncology Unit was used to identify all cases. Patient demographics and oncologic results were recorded. Functional assessment was performed using the Musculoskeletal Tumor Society rating scale and the Toronto Extremity Salvage Score.

*Results*. Nine tumours were about the knee and three were in the proximal humerus. Negative margins were achieved in all cases. No patient had metastatic disease at a mean follow-up of 56.5 months. There was one local recurrence and this was managed by conversion to a Van Nes rotationplasty. Functional results were excellent in the proximal humeral cases and in cases about the knee where the stabilizing ligaments were preserved. Cases with post-operative knee instability were less successful but none the less were well controlled with bracing.

*Discussion*. This technique offers an alternative to joint excision and prosthetic replacement in a group of young patients.


Sarcoma (1997) 1, 167–174

CARFAX

ORIGINAL ARTICLE

### The geometric osteotomy: joint preservation in juxta-articular surface bone neoplasms

ERIC L. MASTERSON,[1] RICCARDO FERRACINI,[1] AILEEN M. DAVIS,[1,2] JAY S. WUNDER[1,2] & ROBERT S. BELL[1,2,3]

[1]*University Musculoskeletal Oncology Unit, Mount Sinai Hospital,* [2]*University of Toronto &* [3]*Princess Margaret Comprehensive Cancer Centre, Toronto, Canada*

**Abstract**

*Purpose.* To present the oncologic and functional results of a consecutive series of patients treated by geometric osteotomy and allograft reconstruction for juxta-articular surface bone neoplasms.

*Patients.* Twelve consecutive patients (mean age 28 years) underwent excision of benign aggressive or malignant juxta-articular surface bone neoplasms. In each case, only part of the circumference of the bone was excised, and the joint surface was preserved and kept in continuity with the remainder of the bone. The defects were filled with allograft or autograft, and internally fixed as appropriate.

*Methods.* Patients were eligible for the study if they had a histologically proven, primary tumour of bone adjacent to a joint such that the tumour could be completely excised with a partial cortical resection and preservation of the joint. The database at the University Musculoskeletal Oncology Unit was used to identify all cases. Patient demographics and oncologic results were recorded. Functional assessment was performed using the Musculoskeletal Tumor Society rating scale and the Toronto Extremity Salvage Score.

*Results.* Nine tumours were about the knee and three were in the proximal humerus. Negative margins were achieved in all cases. No patient had metastatic disease at a mean follow-up of 56.5 months. There was one local recurrence and this was managed by conversion to a Van Nes rotationplasty. Functional results were excellent in the proximal humeral cases and in cases about the knee where the stabilizing ligaments were preserved. Cases with post-operative knee instability were less successful but none the less were well controlled with bracing.

*Discussion.* This technique offers an alternative to joint excision and prosthetic replacement in a group of young patients.

**Key words:** *geometric osteotomy, joint preservation, allograft, juxta-articular surface bone neoplasms.*

### Introduction

Primary bone neoplasms occurring in the metaphysis of a long bone commonly have extensive involvement of the medullary canal. For this reason, adequate excision during limb-salvage surgery usually necessitates excision of the entire metaphysis and adjacent joint, followed by reconstruction by arthrodesis, with a tumour prosthesis, an allograft or a combination of both.

Less commonly, a malignant or benign aggressive neoplasm may arise *de novo* from the surface of a long bone and the medullary canal may be normal or only minimally involved. Examples of these tumours include periosteal osteosarcoma, parosteal osteosarcoma, periosteal chondroma and juxta-cortical chondrosarcoma. Surface malignancies tend to be less aggressive than those with extensive medullary involvement, although a significant percentage of parosteal osteosarcomas may have histological evidence of dedifferentiation.[1] Surface neoplasms typically occur in adults before middle age and the most common locations are the distal femur, proximal tibia and proximal humerus. They are commonly managed by wide resection of the metaphysis and the adjacent joint with subsequent reconstruction.[2,3] Periosteal chondromas may recur following intralesional excision and should, if possible, be treated by marginal resection.[4]

Reconstruction of the knee joint following resection is most commonly performed with commercially available tumour prostheses, osteoarticular allografts, arthrodesis, or a combination of allograft and implant. Each of these options has potential disadvantages. Prostheses may suffer from a host of difficulties, including extensor mechanism problems, infection, failure of polyethylene components and, with time, stem fracture or aseptic loos-

Correspondence to: R. S. Bell, University Musculoskeletal Oncology Unit, Mount Sinai Hospital, 600 University Avenue, Suite 476, Toronto, Canada M5G 1X5. Tel: + 1 416 586 8607; Fax: + 1 416 586 8397; E-mail: bell@mshri.on.ca.

1357-714X/97/030167–08 $9.00 © 1997 Carfax Publishing Ltd ening. Allografts may become infected or fracture and ligamentous instability is not uncommon.

Shoulder reconstruction following resection of the proximal humerus may be accomplished with a tumour prosthesis, an ostechondral allograft or an intercalary arthrodesis. Again, each of these options suffers from a range of drawbacks and potential complications.[5,6]

The advent of accurate cross-sectional imaging by computed tomography (CT) and magnetic resonance imaging (MRI) has permitted surgeons to determine accurately the extent of cortical attachment and the presence or absence of medullary involvement of these surface neoplasms. Our group has taken advantage of this information to plan and perform a series of tumour resections for these juxta-cortical lesions while preserving the continuity of the bone and the adjacent articular surfaces. The term 'geometric osteotomy' has been used to describe this approach[7] and this paper presents our experience of this procedure for the management of juxta-articular surface malignant or benign aggressive tumours.

### Patients and methods

Patients were eligible for this study if they had a juxta-cortical, primary bone tumour adjacent to a joint such that the tumour could be completely excised with a hemi-cortical resection and reconstructed with allo- or autogenous bone graft. The database maintained at our unit was used to identify all eligible cases treated since 1986.

All patients who present to our unit with suspected surface malignancy of bone are evaluated locally with plain radiography, isotope bone scan, CT and MRI. We pay particular attention to the width of the cortical attachment, the presence of medullary involvement and the relationship of the tumour to the joint surface. When the imaging studies are complete, we perform an open biopsy to confirm the diagnosis if the radiographic assessment is inconclusive. If the radiographic appearance is absolutely typical and the lesion can be excised completely without major morbidity, we proceed to resection without prior biopsy, as recommended by Enneking *et al.*[2]

On the one hand, if the diagnostic work-up confirms that the cortical attachment of the lesion is not excessively wide and that medullary involvement is absent or minimal, we plan a wide excision of the lesion with preservation of bony continuity and the adjacent articular surface. If, on the other hand, the cross-sectional imaging demonstrates that the bone is effectively encircled by tumour or that there is extensive medullary involvement, we proceed with resection of the entire width of the bone with, if necessary, the adjacent joint. If it is determined that at least 25% of the cortex circumference can be safely maintained while adequately excising the tumour, the patient is considered eligible for a geometric osteotomy.

In planning the geometric osteotomy, each case must of necessity be individualized. However, there are a number of principles which are common to all cases. The biopsy incision is excised with a cuff of normal tissue. The periosteum is incised circumferentially around the tumour leaving a margin of normal tissue as determined by pre-operative planning and intra-operative palpation. Around the knee, the proximity of the lesion to the joint frequently necessitates division of some of the joint capsule and defunctioning of one or more of the stabilizing ligaments of the joint (most commonly the posterior cruciate ligament, PCL). The bone cuts are determined by the shape and dimensions of the tumour and are generally accomplished with a series of drill holes which are connected with cuts performed using an osteotome. The tumour is then removed leaving the contralateral cortex and the joint surfaces intact. The medullary surface of the resection specimen is then evaluated carefully to confirm adequate margins.

Reconstruction is carried out using a block of allograft or iliac crest autograft which is carefully fashioned to fit the contours of the bone defect. Repeated fitting of the allograft is undertaken until an acceptable match of contour has been achieved. The graft is then fixed in place with screws and, most commonly, with a supporting buttress plate to prevent post-operative fracture. When allograft is used, it is often supplemented with corticocancellous chips harvested from the patient's iliac crest in order to promote union.

Finally, the adjacent joint is evaluated to determine stability. If possible, we reconstruct the resected ligaments by direct suture repair or with locally harvested tendon, fascia or muscle transfer. Post-operatively, weight-bearing is protected until the follow-up radiographs show early evidence of union. Most patients are kept partial weight-bearing for at least 6 weeks and then gradually progress to full weight.

For each eligible patient, information was obtained from the pre-operative consultation note and the operation and pathology reports, as well as the pre- and post-operative radiographic studies. Each patient was re-evaluated clinically and radiographically. Active and passive range of motion for the knee or shoulder joint (as appropriate) was noted for each patient, as was the presence or absence of any joint instability in the antero-posterior or medio-lateral planes.

Function was evaluated using the Musculoskeletal Tumor Society (MSTS) rating scale, 1987 and 1993[8,9] versions and the Toronto Extremity Salvage Score (TESS).[10] The 1987 version of the MSTS includes evaluation of clinical parameters such as range of motion, strength, joint stability and joint deformity as well as evaluation of pain, overall functional ability and acceptance of the treatment. The 1993 version of the MSTS maintains the latter three items and evaluates use of ambulatory aid, walking ability and gait handicap. Both versions of the MSTS were used as their content differs. The 1987 version has a maximum score of 35 and the 1993 version is converted to a percentage. The MSTS (1987 and 1993) were scored by a single clinician. The TESS is a measure of physical disability[11] and evaluates, based on the patient's own perception, the difficulty experienced performing various daily tasks and activities. The TESS provides a standardized score ranging from 0 to 100.[10]

### Results

Twelve patients were identified who underwent geometric osteotomies between June 1986 and February 1996 for juxta-articular surface tumours that were considered aggressive or malignant. Details of patient demographics and pre-operative diagnoses are provided in Table 1 (see also Figs 1–4). There were five males and seven females, and the mean age at the time of surgery was 28 years (range 17–41 years). The initial diagnosis was parosteal osteosarcoma (five cases), periosteal chondroma (three cases), chondrosarcoma (three cases) and periosteal osteosarcoma (one case). The location of the lesion was distal femur (eight cases), proximal humerus (three cases) and proximal tibia (one case). All lesions were metaphyseal or epiphyseal–metaphyseal. No patient had evidence of metastatic disease at presentation.

The most common presenting feature was a mass (nine patients). Seven patients complained of pain related to their tumour, four had noticed a reduced range of movement in the adjacent joint and two patients gave a history of recent trauma to the area. Only one patient had undergone previous surgery to the area. This was an intralesional excision of a periosteal chondroma that presented as a recurrence 2 years post-operatively.

In eight patients, the diagnosis was confirmed by open biopsy; in the remaining four patients, the diagnosis was confirmed on pathological examination of an excisional biopsy with wide margins.

### *Surgery*

All bony resections came to within 3 cm of the articular surface and, in seven cases, the resection came to within 1 cm of the joint surface. The defect was filled with a block of allograft in five cases, autograft in two cases and both allograft and autograft in three cases. In two patients, the defect was sufficiently small not to require bone graft. The percentage of the circumference of the bone involved by tumour ranged from 15 to 66% (detailed in each case in Table 1) and the amount of circumferential cortex excised exceeded the extent of cortical involvement. One patient sustained an intra-operative fracture following resection of a low-grade chondrosarcoma of the proximal humeral metaphysis (case number 8). This was plated and grafted and healed uneventfully. Soft tissue reconstructions included two gastrocnemius muscle belly transposition flaps, one reconstruction of the knee joint capsule with marlex mesh, three direct capsular repairs and one reconstruction of the lateral collateral ligament of the knee with a strip of fascia lata. There were no post-operative neurovascular sequelae and all wounds healed without incident.

Histological examination revealed the resection margins to be negative in all cases. The closest documented bone margin was 5 mm from the tumour.

### *Adjuvant therapy*

One patient received adjuvant post-operative chemotherapy using cisplatin and adriamycin for a dedifferentiated parosteal osteosarcoma.

### *Follow-up*

Mean follow-up was 56.5 months (range 9–123 months). Follow-up details are outlined in Table 2. Tumour recurrence was identified in only one patient (case 6). This patient had an *en bloc* excision of a tumour that was diagnosed pathologically as a periosteal chondroma based on a previous curettage but recurred after 15 months. A further recurrence was found 1 year after this second operation and at this second recurrence, the lesion was confirmed as a high-grade dedifferentiated parosteal osteosarcoma. This was treated by wide excision and a Van Nes rotationplasty. The patient remains clinically tumour free 8 years after the latter surgery.

No patient had evidence of metastatic disease at the time of review.

The three patients treated by geometric osteotomy for tumours of the proximal humerus reported no functional deficits or residual symptoms and this was reflected by perfect scores on the MSTS (1987 and 1993) and the TESS (see Table 2).

Nine patients were treated by geometric osteotomy for tumours about the knee joint (eight distal femur, one proximal tibia). Four of these patients complained of mild pain for which non-steroidal anti-inflammatory medications were occasionally used. All reported that medication use depended on activity level. As seen in Table 1, all patients had a minimum of 100° flexion at the knee. All but one patient had decreased quadriceps strength on gross motor testing. Joint stability was problematic in that three patients experience 'giving way'; one requires bracing for daily ambulation and two use a cane. All three of these individuals had

**Table 1.** *Patient demographics and pre-operative details*

| Case | Age (years) | Sex | Side | Diagnosis | Location | Distance from joint | % cortex circumference involved by tumour on CT/MRI | Medullary involvement | Pre-operative biopsy |
|---|---|---|---|---|---|---|---|---|---|
| 1 | 33 | M | R | Periosteal chondroma | Distal femur | 1 | 10 | No | Yes |
| 2 | 26 | F | L | Periosteal osteosarcoma | Distal femur | 1 | 30 | No | Yes |
| 3 | 25 | F | R | Parosteal osteosarcoma | Distal femur | 2 | 35 | No | No |
| 4 | 24 | F | R | Parosteal osteosarcoma | Distal femur | 0 | 30 | No | No |
| 5 | 25 | F | R | Parosteal osteosarcoma | Distal femur | 1.5 | 40 | No | Yes |
| 6 | 19 | M | L | Periosteal chondroma | Distal femur | 1 | 30 | No | Yes |
| 7 | 17 | F | L | Parosteal osteosarcoma | Proximal humerus | 0.5 | 50 | Yes | Yes |
| 8 | 20 | M | R | Periosteal chondroma | Proximal humerus | 3 | 15 | No | Yes |
| 9 | 37 | M | R | Juxta-cortical chondrosarcoma | Proximal tibia | 0.5 | 35 | Yes | Yes |
| 10 | 34 | F | R | Parosteal osteosarcoma | Distal femur | 3 | 25 | No | Yes |
| 11 | 33 | M | L | Juxta-cortical chondrosarcoma | Proximal humerus | 3 | 15 | Yes | No |
| 12 | 41 | F | L | Juxta-cortical chondrosarcoma | Distal femur | 0 | 40 | Yes | No |

**Table 2.** *Surgical procedure and post-operative follow-up*

| Case | Margins | Allograft/ autograft | Internal fixation | Soft tissue reconstruction | Follow-up (months) | Local recurrence | MSTS (1987) | MSTS (1993) | TESS | Range of motion |
|---|---|---|---|---|---|---|---|---|---|---|
| 1 | Negative | Neither | None | Gastrocnemius flap | 116 | No | 29 | 80 | 73 | Full |
| 2 | Negative | Allograft | Plate and screws | Gastrocnemius flap, fascia lata for LCL | 15 | No | 31 | 93 | 97 | Full |
| 3 | Negative | Allograft and autograft | Plate and screws | No | 49 | No | 26 | 67 | 52 | Full* |
| 4 | Negative | Allograft | Plate and screws | No | 13 | No | 35 | 100 | 100 | Full |
| 5 | Negative | Allograft | Plate and screws | No | 123 | No | 32 | 93 | 90 | 0–110 |
| 6 | Negative | Allograft | Plate and screws | No | 15 | Yes | Van Nes | Van Nes | Van Nes | Van Nes |
| 7 | Negative | Autograft | Plate and screws | Capsular repair | 72 | No | 35 | 96 | 99 | Full |
| 8 | Negative | Autograft | Plate and screws | No | 49 | No | 35 | 100 | 100 | Full |
| 9 | Negative | Allograft and autograft | Screws | Capsular repair | 9 | No | 20 | 53 | 75 | 0–130* |
| 10 | Negative | Allograft | Screws | Capsular repair | 30 | No | 27 | 80 | 87 | 0–110 |
| 11 | Negative | Neither | None | No | 73 | No | 35 | 100 | 100 | Full |
| 12 | Negative | Allograft and autograft | Plate and screws | Marlex mesh to capsule | 114 | No | 21 | 80 | 81 | 0–100* |

*Clinical PCL laxity.

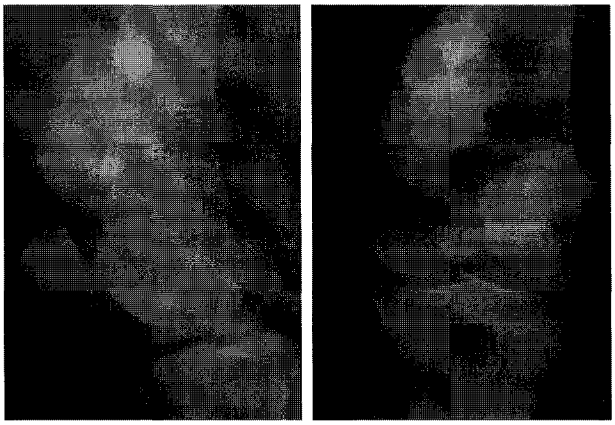

**Fig. 1.** *Twenty-four-year-old female patient with a parosteal osteosarcoma of the right distal femoral metaphysis (case 4).*

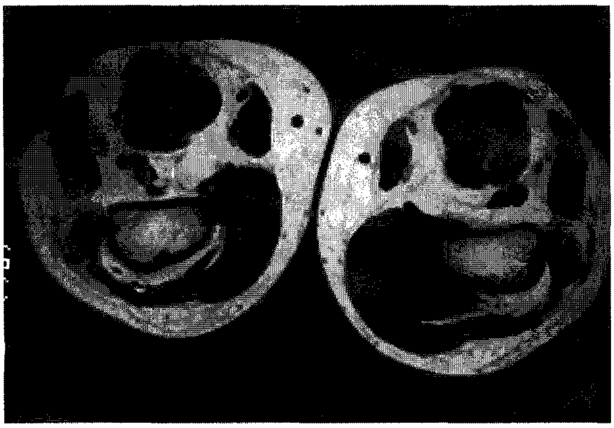

**Fig. 2.** *MRI using T1-weighted sequence showing medial surface tumour without obvious medullary invasion occupying approximately 30% of the circumference of the cortex.*

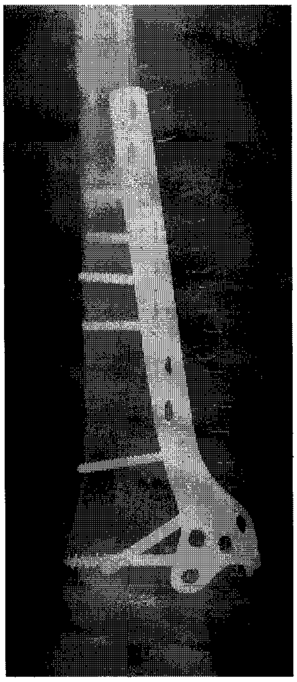

**Fig. 3.** *Immediate post-operative radiograph demonstrating geographic osteotomy and reconstruction with carefully fashioned allograft and plate and screws.*

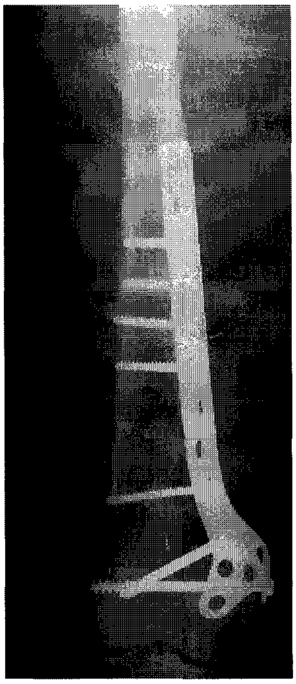

**Fig. 4.** *The same patient 2 years later demonstrating progressive graft incorporation.*

their posterior cruciate ligament resected as part of the tumour excision. Overall function was good, although none of the patients was able to participate in impact sports and all reported difficulty ambulating on uneven terrain. On average, the 1987 MSTS score was 29.57, SD = 5.56, with points most frequently lost for stability pain and overall function.

Overall, the patients who rated themselves as mildly disabled had a mean TESS score of 86.7 (SD = 15.45) following perigenicular tumour excisions. Three had scores of 90 or more and rated themselves overall as 'not at all disabled'. Four had scores in the range 70–89 and rated themselves as mildly disabled. One patient with a score of 52 considered herself to be moderately disabled. This patient was PCL deficient and required a brace and cane for ambulation outdoors.

### Discussion

This small series demonstrates that it is possible to resect juxta-articular surface tumours with adequate margins while still preserving acceptable joint function. We have had only one local recurrence in a patient in whom the tumour had undergone dedifferentiation from a periosteal chondroma to a high-grade parosteal osteosarcoma. The issue of what constitutes an adequate margin for parosteal osteosarcomas has been addressed by Enneking *et al.* in a review of their experience with this uncommon tumour.[2] They concluded that while the risk of recurrence following a wide excision was negligible, low recurrence rates could also be achieved by well planned marginal resections. They noted 100% recurrence following intralesional excision but considered radical resections to be unnecessary. The appropriate resection must lie between these two extremes. The appropriate resection margins for juxta-cortical chondrosarcoma of low or intermediate grade can be presumed to be similar as the pattern of recurrence is similar.

Okada *et al.*, in a review of the Mayo Clinic experience of parosteal osteosarcoma, noted very high recurrence rates following marginal excision.[1] The latter was defined as a soft tissue margin of less than 3 mm and a bony margin of less than 2 cm. By this definition, all but one of the resections described in this article were marginal. However, it should be noted that the majority of the patients in the Mayo Clinic series were treated prior to the routine use of CT or MRI. These modalities have added greatly to the accuracy of pre-operative planning by determining the extent of cortical attachment and the presence of significant medullary involvement. This has allowed us to avoid attempting geometric osteotomy in cases that would be more appropriately dealt with by a wider excision.

The principal advantage of the geometric osteotomy is that the patient retains his or her own joint. When the alternative surgical options are considered, the geometric osteotomy is particularly advantageous in the young adult population in whom these lesions tend to occur. Arthrodesis with an intercalary bone graft has been used following wide excision of the tumour and adjacent joint.[5,6,12] While this does provide a long-term solution, there may be considerable difficulty in achieving a solid fusion and patient satisfaction with arthrodesis can be limited.

Wide excision and reconstruction with a tumour prosthesis has also been used for these tumours.[3,13,14] This obviates the disadvantages of a stiff knee but introduces a range of new potential complications. In addition, most of these patients are young adults and the risk of early aseptic loosening of a prosthesis with associated bone loss is considerable.

There are disadvantages to a geometric osteotomy. The proximity of the joint means that, of necessity, joint capsule and stabilizing ligaments sometimes need to be excised in order to achieve negative margins. This may cause symptomatic joint instability and predispose the patient to the development of premature osteoarthritis. However, we feel that any instability can be adequately managed by external bracing. Even if osteoarthritis develops in the long term in some cases, the delay in coming to a total joint arthroplasty will have been worthwhile given the limited life expectancy of an arthroplasty in the young adult patient.

### References

1 Okada K, Frassica F, Sim F, *et al.* Parosteal osteosarcoma. A clinicopathological study. *J Bone Joint Surg* 1994; 76-A:366–78.

2 Enneking WF, Springfield D, Gross M. The surgical treatment of parosteal osteosarcoma in long bones. *J Bone Joint Surg* 1985; 67-A:125–35.

3 Kavanagh TG, Cannon SR, Pringle J, *et al.* Parosteal osteosarcoma. Treatment by wide resection and prosthetic replacement. *J Bone Joint Surg* 1990; 72-B:959–65.

4 Boriani S, Bacchini P, Bertoni F, *et al.* Periosteal chondroma. A review of twenty cases. *J Bone Joint Surg* 1983; 65A:205–12.

5 Alman BA, DeBari A, Krajbich JI. Massive allografts in the treatment of osteosarcoma and Ewing sarcoma in children and adolescents. *J Bone Joint Surg* 1995; 77A:54–64.

6 O'Connor MI, Sim FH, Chao EY. Limb salvage for neoplasms of the shoulder girdle. Intermediate reconstructive and functional results. *J Bone Joint Surg* 1996; 78A:1872–88.

7 Czitrom AA. Allograft reconstruction after tumor surgery in the appendicular skeleton. In: Gross AE, Czitrom AA, eds. *Allografts in orthopaedic practice.* Baltimore, MD: Williams & Wilkins, 1992:83–119.

8 Enneking WF. Modification of the system for functional evaluation in the surgical management of musculoskeletal tumours. In: Enneking WF, ed. *Limb salvage in musculoskeletal oncology.* New York: Churchill Livingstone, 1987:626–39.

9 Enneking WF, Dunham W, Gebhardt MC, *et al.* A system for the functional evaluation of reconstructive procedures after surgical treatment of tumors of the musculoskeletal system. *Clin Orthop Rel Res* 1993; 286:241–6.

10 Davis AM, Wright JG, Williams JI, *et al.* Development of a measure of physical function for patients with bone and soft tissue sarcomas. *Quality of Life Research* 1996; 5(5):508–16.

11 World Health Organization. *International classification of impairments, disabilities and handicaps.* Geneva: WHO, 1980.

12 Weiner SD, Scarborough M, Vander Griend RA. Resection arthrodesis of the knee with intercalary allograft. *J Bone Joint Surg* 1996; 78A:185–92.

13 Nixon JE. Prostheses in the management of bone cancer. *Br Med J* 1983; 287:245–6.

14 Sweetman R. Limb preservation in the treatment of bone tumours. *Ann R Coll Surg Eng* 1983; 65:3–7.
